# Oligosaccharide Chromatographic Techniques for Quantitation of Structural Process-Related Impurities in Heparin Resulting From 2-O Desulfation

**DOI:** 10.3389/fmed.2018.00346

**Published:** 2018-12-18

**Authors:** Pascal Anger, Céline Martinez, Pierre Mourier, Christian Viskov

**Affiliations:** ^1^Sanofi, Antony, France; ^2^Aspen NDB, Notre Dame de Bondeville, France

**Keywords:** heparin, process, desulfation, impurities, pharmacopeias, chromatography

## Abstract

Heparin is a widely-used intravenous anticoagulant comprising a complex mixture of highly-sulfated linear polysaccharides of repeating sequences of uronic acids (either iduronic or glucuronic) 1->4 linked to D-glucosamine with specific sulfation patterns. Preparation of crude heparin from mammalian mucosa involves protease digestion with alcalase under basic conditions (pH ≥ 9) and high temperature (>50°C) and also oxidation. Under such conditions, side reactions including the ubiquitous 2-O desulfation occur on the heparin backbone yielding non-endogenous disaccharides within polysaccharide chains. Whatever the process used for its manufacture, some level of corresponding degradation impurities is therefore expected to be found in heparin and the derived Low Molecular Weight Heparins. These impurities should be monitored to control the quality of the final therapeutic product. Two anion exchange chromatography techniques were used to analyze heparin samples exhaustively or partially depolymerized with heparinases and determine the proportions of non-endogenous disaccharides generated by side reactions during the manufacturing process (epoxides and galacturonic moieties). We also present data from a case study of marketed heparin. Current heparin sodium monographs do not directly address process impurities related to modification of the structure of heparin. Although desulfation reduces the overall biological potency, we found that heparin with an average of one modified disaccharide per chain can still comply with the USP or Ph. Eur. heparin sodium monographs requirements. We have implemented disaccharide analysis to monitor the quality of this product on a risk basis.

## Introduction

Heparin is a key pharmaceutical product in the thrombosis field. It can be used therapeutically as unfractionated heparin (UFH), or as the starting material in the preparation of low-molecular-weight heparins (LMWHs). UFHs are highly polydisperse mixtures of glycosaminoglycans (GAGs), with a typical average molecular weight of 15,000 Da. Its anticoagulant potency is mostly expressed in anti-factor IIa activity (anti-FIIa), which must be at least 180 U/mg according to the United States Pharmacopeia (USP) and European Pharmacopoeia (Ph. Eur.) monographs (1, 2).

The purification processes from mucosa to pure heparin can usually be divided into three stages: (1) processing from mucosa to small batches of raw heparin; (2) consolidation of the small batches of raw heparin into crude heparin batches of variable size and; (3) purification from crude to pure heparin. The first stage often involves stabilization of mucosa with sodium metabisulfite, followed by alcalase depolymerization of heparin proteoglycan under basic conditions (pH ≥ 9) and high temperature (>50°C). Stage 3 of the heparin purification processes also involve basic conditions, with high temperatures (>50°C) and different types of oxidation. Cationic resins or alkoxy ammonium precipitating agents and several steps of alcoholic precipitation are used in these process stages to isolate heparin from many impurities such as proteins, nucleic acids, and related GAGs (mainly dermatan sulfate and heparan sulfate).

The complexity of the supply chain in terms of geographical areas, number of raw heparin processing plants, and manufacturing processes raises potential concerns about the quality of heparin products at upstream stages due to the high number of contributors. Sanofi processes mucosa and crude heparin from different suppliers and therefore has a broad overview of the different manufacturing processes and consequently of the associated potential impurities.

The different USP and Ph. Eur. monographs upgrades have dramatically improved the control of heparin quality but yet do not address directly process impurities derived from the heparin structure, i.e., the structural modification on the polysaccharide backbone. Instead the monograph specifications indirectly rely on increased activity requirement (anti-FIIa ≥180 U/mg for USP and Ph. Eur.) as a surrogate marker for structural integrity, which makes the accuracy and reproducibility of activity measurement crucial (3). As of July 2014, the Ph. Eur. has moved from requiring anticoagulant (anti-coag) to anti-FIIa activity measurements without modifying the specification of 180 U/mg. Although not always the case, it can be expected that most degradation reactions during the manufacturing process will alter antithrombin (AT) binding sites and ultimately induce some potency decrease. However, the acceptability of marginally degraded, although compliant, products may be questioned. Side reactions do not only occur around AT-binding sites and structural modifications that do not modify anti-FIIa activity may still have an impact on other biological properties of heparin (protein binding, pK, etc.). Therefore, relying only on anti-FIIa or anti-coag activity may not be sufficient to capture the different modifications of the macromolecular backbone and ultimately control their potential biological effects.

Although different process structural impurities (4–7) impacting or not anti-IIa are known and either systematically present or present at sometimes significant levels, we here only focus on those resulting from the ubiquitous 2O-desulfation. We review this key side reaction and discuss how disaccharide and oligosaccharide analyses using strong anion-exchange (SAX) chromatography after heparinase digestions can be used to monitor these. We further present the findings of an investigation of these structural modifications undertaken as part of the follow-up on an out of specification (OOS) heparin batch. The discussed techniques can be used to further improve quality control at the raw/crude/pure stages of heparin production.

## Materials and Methods

### Reagents and Chemicals

Sodium perchlorate (NaClO_4_), monobasic sodium phosphate (NaH_2_PO_4_), calcium acetate (Ca[C_2_H_3_O_2_]_2_), and monobasic potassium phosphate (KH_2_PO_4_) were purchased from VWR/Prolabo (Lutterworth, UK). Acetic acid (C_2_H_4_O_2_) and bovine serum albumin (BSA) were obtained from Acros Organics (Geel, Belgium) and Sigma-Aldrich (St. Louis, MO, USA), respectively. Sodium hydroxide (NaOH) and potassium hydroxide (KOH) were purchased from Chemlab (Essex, UK). Phosphoric acid (H_3_PO_4_) was obtained from Mallinckrodt (Phillipsburg, NJ, USA). All enzyme lyases from Flavobacterium heparinum (heparinase 1 [EC 4.2.2.7], heparinase 2 [no EC number], and heparinase 3 [EC 4.2.2.8]) were purchased from Grampian Enzymes (Aberdeen, United Kingdom). All other reagents and chemicals were of the highest quality available. Water was purified using a Millipore purification system (Guyancourt, France). Factor IIa and human AT were obtained from Stago (Asnières-sur-Seine, France). Factor Xa was of bovine origin and Factor IIa was of human origin. Enzymes and substrates [S2765 for Factor Xa and S2238 for Factor IIa] were obtained from Chromogenix (Paris, France). The USP heparin standard solution was used for anti-FXa and anti-FIIa activity measurements; Ph. Eur. standard solutions were used for anti-coag activity measurements. Ninety-six-well deep-well microplates for anti-FXa and anti-FIIa assays were obtained from Greiner Bio-One (Paris, France).

### Disaccharide Analysis

#### Heparinase Digestions

Exhaustive depolymerization of heparin was performed at room temperature for 48 h in a total volume of 190 μL containing 20 μL of 20 mg/mL heparin solution in water, 70 μL acetate solution pH 7.0 (2 mM Ca[C_2_H_3_O_2_]_2_ and 0.1 mg/mL BSA), and 100 μL heparinases 1+2+3 solution containing 0.13 IU/mL of each heparinase in a pH 7.0 potassium phosphate buffer (10 mM KH_2_PO_4_ and 0.2 mg/mL of BSA).

Partial digestion of heparin with heparinase 1 was performed at 16°C for 48 h with 10 mIU heparinase 1 in a total volume of 130 μL containing 20 μL of 20 mg/mL heparin solution in water, 90 μL 5 mM NaH_2_PO_4_, pH 7.0, containing 200 mM NaCl and 2 mg/mL BSA.

### Analysis of Heparin Digests With SAX Chromatography

The comparative disaccharide analysis was performed using a gradient high-performance liquid chromatography (HPLC; Agilent 1100, Cheshire, UK) in SAX mode. Digested heparin solution (8 μL) was injected onto a Spherisorb SAX 250 × 3.2 mm, 5 μm column (Waters, Saint-Quentin en Yvelines, France). The column temperature was set at 50°C, the flow rate to 0.45 mL/min, and UV detection was carried out at 234 nm. Solvent A was 1.8 mM NaH_2_PO_4_ at pH 3.0, and solvent B was an aqueous solution of 1.8 mM NaH_2_PO_4_ with 1 M NaClO_4_ adjusted to pH 3.0. A 50-min solvent B gradient with different slopes, starting from 3 to 100%, was used and the highest solvent B% flow was held for 10 min. The integration was performed with Empower® (Waters, Saint-Quentin en Yvelines, France). Depolymerisation, HPLC, and quantification were performed as previously described (4). These procedures can also be found in the USP general chapter <207> or Ph. Eur. Enoxaparin monograph (8, 9).

### Analysis of Heparin Digests With Cetyltrimethylammonium (CTA)-SAX Chromatography

The analysis of heparin digested by heparinase 1 was performed with cetyltrimethylammonium (CTA)-SAX chromatography as described previously (10). Briefly, 150 × 2.1 mm columns filled with Kinetex C_18_ 1.7 μm particles (Phenomenex, Le Pecq, France) were dynamically coated using CTA using 1 mM CTA HSO_4_ in H_2_O-CH_3_OH (65:35 v/v) as the solvent for equilibration. Eluents used for CTA-SAX chromatography were as previously described (10). A linear concentration gradient (0–2 M) of aqueous ammonium methane sulfonate, pH 2.5 was applied. Flow rate was set at 0.35 mL/min. Column temperature was 40°C. Double UV detection was performed as described previously at 232 nm and at 202–242 nm. The N-acetylated oligosaccharide selective signal, 202–242 nm, is determined by subtracting the absorbance at 242 nm (reference signal) from that at 202 nm (detection wavelength).

### Analysis of Anti-coag, Anti-factor IIa, and Anti-factor Xa Activities

Anti-coag activity, anti-factor IIa activity (anti-FIIa), and anti-factor Xa activity (anti-FXa) were performed according to the USP general chapter <208> and Ph. Eur. assay of heparin related to UFH (11, 12). Concentrations of standard and sample were assessed in duplicate. Three independent experiments were performed for each assay. All dilutions, dispensing, mixing, and control of incubation times were performed by the Microlab Star platform from Hamilton (Villebon sur Yvette, France). UV reading at end point was performed using EMax detector from Molecular Device (Sunnyvale, CA, USA). Statistical analysis was performed according to the Ph. Eur. § 5.3 for parallel-line models (13). All activities were expressed on dry basis.

### Measurement of Mass Average Molecular Weight

The mass average molecular weight (MW) was obtained by size-exclusion chromatography (SEC). Each standard and sample (20 μL) was injected onto two columns (TSKgel 5 μm G3000SWXL and G2000SWXL 300 × 7.8 mm) used in series (TOSOH Biosciences, Lyon, France). Mobile phase was 0.5 M lithium nitrate (LiNO_3_), flow rate 0.6 mL/min, temperature 30°C, and refractive index detection was used. Calibration was performed using a broad standard table associated with the internally-qualified standard, which is similar to the procedure described in the current USP Heparin Sodium monograph (1).

## Results and Discussion

### Degradation of Heparin in Basic Conditions

**Figure 1:**
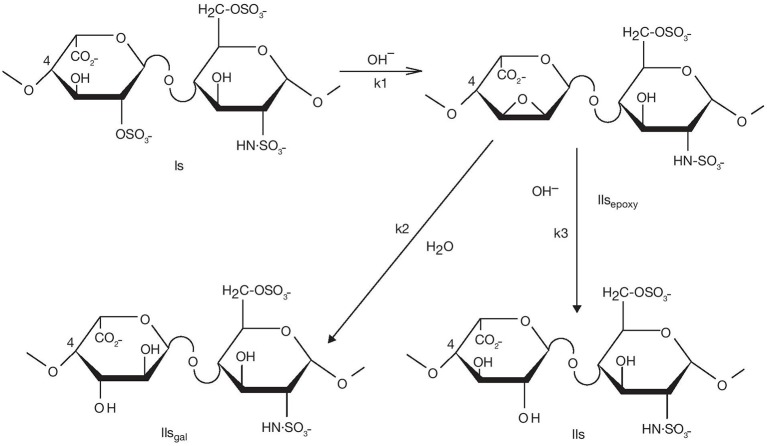
Desulfation of the Is disaccharide in basic conditions.

The degradation mechanism of heparinoids in basic conditions was studied in depth to characterize the LMWH enoxaparin. In the enoxaparin purification process, modification of the heparin endogenous backbone occurs during depolymerization of the heparin ester when the heparin benzyl ester is heated in presence of NaOH. Mechanistically, the cleavage of the heparin ester chain is the result of two competing chemical reactions, β**-**elimination and ester hydrolysis. Three key side reactions can occur after the β-elimination, caused by the alkalinity of the media: C-2 epimerization of the reducing end glucosamine to mannosamine in alkaline media (14, 15); 6-O desulfation with the formation of a 1,6-anhydro ring at the reducing end; and 2-O desulfation of uronic acids. The first two side reactions observed during enoxaparin purification, while theoretically possible, are normally marginal for heparin due to the low amount of reducing ends available. However, 2-O desulfation of uronic acids has been reported as one of the main mechanisms of heparin degradation (16, 17). Although minor amounts of 2-O sulfated glucuronic acids have been previously reported in heparin (18), the vast majority of 2-O sulfated uronic acids have an iduronic configuration. An epoxy intermediate compound also obtained during 2-O desulfation further hydrolyses into either an iduronic acid or a galacturonic acid (Figure 1).

Desulfation can occur at all disaccharides with a 2-O-sulfated uronic acid moiety, i.e., Is, IIIs, Ia, and IIIa. However, as their typical weight percentages (w/w%) in heparin are 60, 6, 1, and 1% respectively, only IIs_gal_ and IVs_gal_ are readily identifiable by chromatographic analysis. Due to its abundance, we will focus more on IIs saccharides derived from Is, which comprise the natural disaccharide IIs and the two non-endogenous disaccharides IIs_gal_ and IIs_epoxy_ (Figure 1). Nevertheless, we will also give some information on IVs_gal_ formation.

### Quantitation of the Overall 2-O Desulfation Phenomenon

Analytical quantitation of the overall 2-O desulfation is not straightforward.

**Figure 2:**
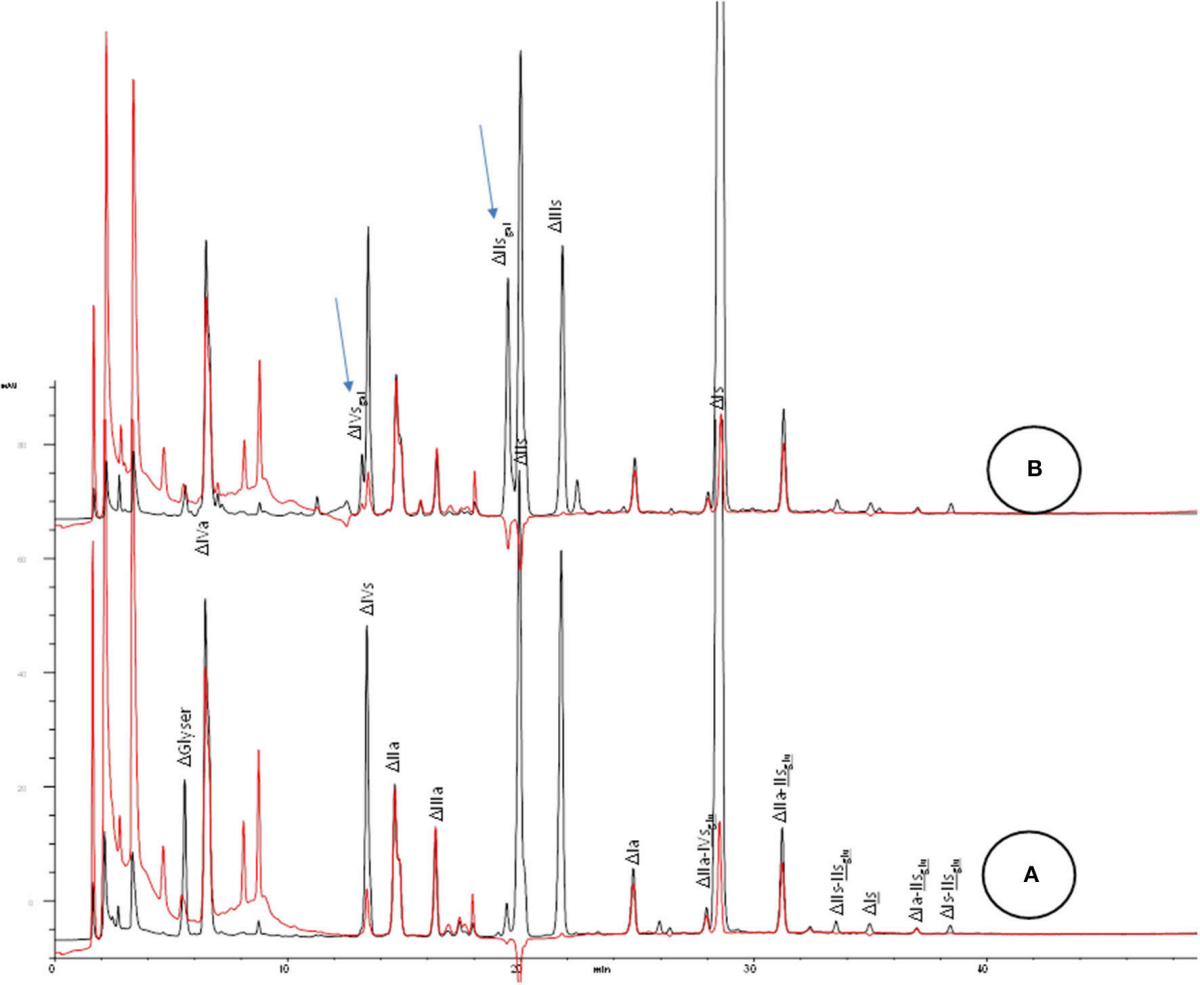
SAX analysis of crude heparin batches after heparinase 1+2+3 depolymerisation for **(A)** typical heparin; **(B)** partially 2-O desulfated heparin. Black line: UV 234 nm; red line: UV 202 nm-242 nm. Arrows for assumed desulfated oligosaccharides.

Chromatographic analysis of the disaccharides generated after exhaustive depolymerisation by heparinases mixture allows the monitoring of two main markers of 2-O desulfation i.e., ΔIIs_gal_ and ΔIVs_gal_ (Figure 2). This analysis is straightforward and should be implemented first when investigating potential degradation of heparin under basic conditions. An appropriate method is available (4, 8, 9). IIs is also generated, and adds to the already significant endogenous IIs content in heparin (~10%) (Figure 1).

Epoxide disaccharides cannot be assessed after exhaustive depolymerisation by heparinase mixture as this non-endogenous derivative is poorly recognized by heparinases and the disaccharide bearing the epoxy is very unstable. It is actually rapidly degraded after a probable mono-desulfation.

In a partially 2-O desulfated heparin, the –GlcNS6S ↓ IIs_epoxy_ site cannot be cleaved by heparinase 1. When heparinase 2 is used, the cleavage becomes possible, but has a slow reaction kinetic. Heparinase 3 has no action.

Bovine lung heparin is more homogeneous and more 2-O sulfated than porcine intestinal mucosa heparin and thus easier to study. In previous experiments, incomplete depolymerisation was obtained when partially 2-O desulfated (30 and 60 min in 1N NaOH, 60°C) bovine lung heparin was exposed to the heparinase mixture. Although tetrasaccharides ΔIIs_gal_-IIs_epoxy_ and ΔIIs_epoxy_-IIs_epoxy_ were identified (unpublished results), the disaccharide ΔIIs_epoxy_ was not detected. If the same 2-O desulfated heparin is treated by heparinase 1 alone, consecutive epoxidized disaccharides (IIs_epoxy_)_n_ sequences are resistant to depolymerization. Gel permeation chromatography (GPC) subsequently reveals a mixture of oligosaccharides ranging from disaccharides to oligosaccharides larger than dodecasaccharides (in comparison, when typical porcine heparin is depolymerized with heparinase 1, the largest observable hexasaccharide is the truncated AT binding site ΔIs-IIa_id_*-*IIs_glu_).

The main tetrasaccharides generated during heparinase 1 digestion, ΔIs-IIs_gal_ and ΔIs-IIs_epoxy_, have been purified and identified (10). The structure of the most predominant hexasaccharide ΔIs-IIs_epoxy_-IIs_epoxy_ was obtained by heparinase sequencing. When ΔIs-IIs_epoxy_ is exposed to heparinase 2, only ΔIs is obtained. When ΔIs-IIs_epoxy_-IIs_epoxy_ is treated by heparinase 2, only a mixture of ΔIs, ΔIs-IIs_epoxy_ and ΔIIs_epoxy_-IIs_epoxy_ is obtained. These data suggest that the ΔIIs_epoxy_ disaccharide may be either unstable or degraded by heparinase 2, and thereby cannot be quantified directly.

2D-NMR can be used to quantitate epoxidation in heparinoid products (19) and has been successfully applied to crude heparins (20). However, this analytical method cannot be considered a routine technique for a Quality Control laboratory.

As both 2D-NMR and disaccharide analysis after exhaustive depolymerisation have limitations to assess the overall 2-O desulfation pattern in crude heparin, we have developed the following additional chromatographic analysis (10).

### Quantitation of IIs_epoxy_ After Partial Depolymerisation

#### Analysis of Heparinase 1 Digests With CTA-SAX Chromatography

**Figure 3:**
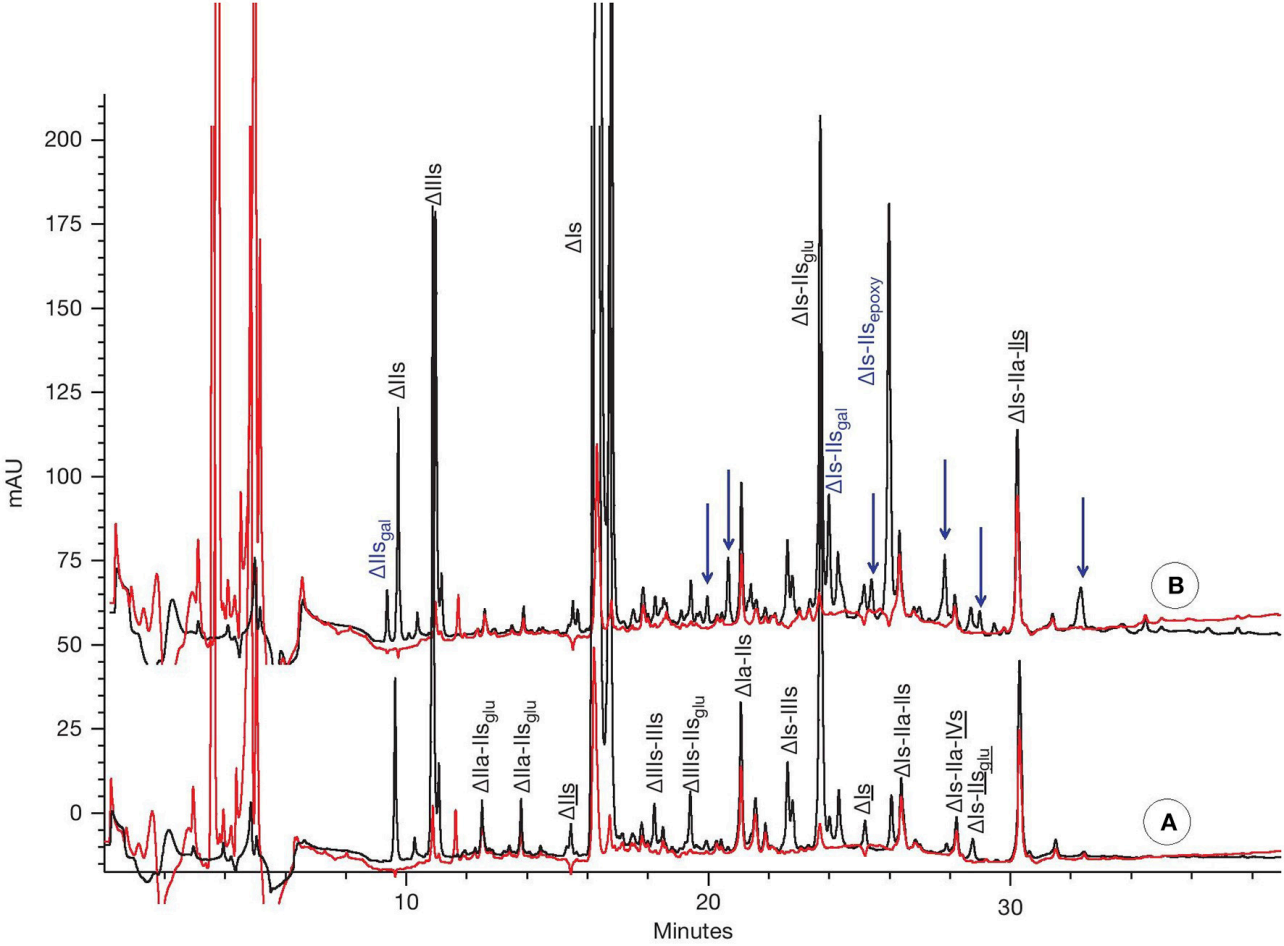
CTA-SAX analysis of heparin batches after heparinase 1 depolymerisation for **(A)** typical heparin; **(B)** partially 2-O desulfated heparin. Black line: UV 234 nm; red line: UV 202–242 nm. Arrows for assumed desulfated oligosaccharides.

As indicated previously, the major components of the mixture obtained after heparinase 1 digestion of porcine mucosa heparin have already been determined. However, the complexity of the mixture obtained makes a quantitative assay of all the components very challenging. In this quantitation method, specifically developed for porcine mucosa heparin, sufficient amounts of heparinase 1 is added to bring the reaction to completion. The resulting digest is a heterogeneous mixture of disaccharides, tetrasaccharides, and hexasaccharides (Figure 3).

Two chromatograms corresponding to heparinase 1 digests of a typical crude heparin (100101) and a partially desulfated crude heparin (9302) differing by their level of 2-O desulfation are shown in Figures 3A,B, respectively. The additional peaks in the chromatogram corresponding to the partially desulfated heparin (Figure 3B) are highlighted with arrows and can be attributed to saccharidic moieties transformed by NaOH, in addition to the well-characterized (desulfated) ΔIs-IIs_epoxy_ and ΔIs-IIs_gal_ moieties.

Although ΔIs-IIs_epoxy_ and ΔIs-IIs_gal_ are the most important oligosaccharides containing IIs_epoxy_ and IIs_gal_, just using the absolute levels of these identified molecules would significantly underestimate the overall levels of the desulfated saccharides and therefore overall degradation. Nevertheless, we can select ΔIs-IIs_epoxy_ and ΔIs-IIs_gal_ as good potential markers to estimate IIs_epoxy_. In our conditions, IIs_gal_ in ΔIs-IIs_gal_ represented on average more than 40% of the overall IIs_gal_ as obtained by depolymerization of heparin with heparinase 1+2+3, thereby confirming the value of this marker for use in quantitation.

#### Calculation of IIs_epoxy_

We here evaluate the entire content of IIs_epoxy_ using:

- the 2 previously mentioned main markers ΔIs-IIs_epoxy_ and ΔIs-IIs_gal_ easily obtained after heparinase 1 digestion
- ΔIIs_gal_ obtained after exhaustive depolymerisation (heparinases 1 +2 +3); this analysis is well-established as quantitation for the entire content of IIs_gal_ (or IIs_gal_)

We then make the assumption that the ratio k of the entire content of IIs_epoxy_ (from the different desulfated species seen Figure 3B) to IIs_epoxy_ in ΔIs-IIs_epoxy_ is the same as that of the entire content of IIs_gal_ (from the different desulfated species seen Figure 3B) to IIs_gal_ in ΔIs-IIs_gal_. The validity of this assumption is limited by the differences of depolymerisation kinetics by heparinase 1 between IIs_gal_ and IIs_epoxy_. The resistance of IIs_gal_ to depolymerization by heparinase 1 is similar to that of IIs_glu_, so that 5–10% of their total amount are already present as disaccharides (Figure 3B). IIs_epoxy_ moieties generate higher resistance to depolymerization, so that the mean molecular weight of oligosaccharides containing IIs_epoxy_ is higher than with IIs_gal_ which could led to different proportions of ΔIs-IIs_epoxy_ compared to ΔIs-IIs_gal_. From heparinase 1 digestion we have:

(1) $$\begin{array}{rcl} \text{entire content of II}{\text{s}}_{\text{epoxy}}\left(or\;IIs_{\text{epoxy}}\right) & = & k\;\times \;IIs_{\text{epoxy}}\;in\;\Delta Is-IIs_{\text{epoxy}}\\ \text{ and entire content of II}{\text{s}}_{\text{gal}}\;\left(\text{or II}{\text{s}}_{\text{gal}}\right) & = & k\;\times \;IIs_{\text{gal}}\;in\;\Delta Is-IIs_{\text{gal}} \end{array}$$

As IIs_epoxy_ and IIs_gal_ have similar molecular weights, we can write the mass contents of IIs_epoxy_ in ΔIs-IIs_epoxy_ and IIs_gal_ in ΔIs-IIs_gal_ as m × ΔIs-IIs_epoxy_ and m x ΔIs-IIs_gal_ respectively. Rearranging (1), we have:

(2) $$\begin{array}{r} k=\frac{IIs_{epoxy}}{IIs_{epoxy}\;in\;\Delta Is-IIs_{epoxy}}=\frac{IIs_{gal}}{IIs_{gal}\;in\;\Delta Is-IIs_{gal}} \end{array}$$

and then

(3) $$\begin{array}{rcl} \;k & = & \frac{IIs_{{}_{epoxy}}\;}{m\;x\;\Delta Is-IIs_{epoxy}}=\frac{IIs_{gal}}{m\;x\;\Delta Is-IIs_{gal}} \end{array}$$

or

$$\begin{array}{l} \frac{IIs_{epoxy}\;}{\Delta Is-IIs_{epoxy}}=\frac{IIs_{gal}}{\Delta Is-IIs_{gal}} \end{array}$$

As indicated above, IIs_gal_ is obtained from (heparinases 1 + 2 + 3) digestion whereas ΔIs-IIs_epoxy_ and ΔIs-IIs_gal_ are obtained from heparinase 1 digestion.

The entire content of IIs_epoxy_ (w/w% in heparin) can therefore be estimated as follows:

$$\begin{array}{l} IIs_{\text{epoxy}}w/w\%=\left(\Delta IIs_{\text{gal}}\;by\;heparinases1+2+3\right)w/w\%\;\\ \times \left(\frac{\Delta Is\;IIs_{epoxy}\;}{\Delta IsIIs_{gal}}\;by\;heparinase1\right). \end{array}$$

### Other Parameters Impacted by Heparin Degradation Under Basic Conditions

The degradation of heparin impacts the activity (anti-coag, anti-IIa, anti-Xa) and the MW of heparin. It has been shown that 2-O desulfation of the Is disaccharide from the AT pentasaccharide binding sequence IIa_id_IIs_glu_Is_id_ results in a complete loss of activity (21). The different activities (anti-FIIa, anti-FXa, and anti-coag) were measured using the methods currently described in the USP general chapter 208 and Ph. Eur. assay of heparin (11, 12). MW characterization was performed with a method similar to that described in the USP heparin sodium monograph (1)—except for the standard, which was not available at the time of study.

### Supplier Case Study

#### Background

Our company purchases crude heparin material from several suppliers to manufacture pure heparin, which is then tested for compliance with current USP and Ph. Eur. specifications. The purification of heparin is performed using batches from a single crude heparin supplier so that pure heparin batches remain traceable to a single supplier. Pure heparin batch 5720 manufactured from crude heparin from supplier A was found to be compliant with the Ph. Eur. heparin sodium monograph for all tests except anti-coag activity (177 U/mg; out of specification of ≥180 U/mg); anti-coag activity was the compendial test at the time of the finding. Measurement of IIs_gal_ and IVs_gal_ led to an estimate of at least 7% 2-O desulfation (ΔIIs_gal_ + ΔIVs_gal_) in this sample (Table 1). To investigate further whether the loss of activity could be linked to the magnitude of 2-O desulfation reactions, we applied our previously described analytical methodology to other heparin batches. Due to this OOS issue, we undertook an in-depth investigation on the batches from supplier A.

**Table 1:** Pure heparin supplier A; disaccharide composition, MW, activities and compliance to the monographs.

**Batch #**	**3966**	**3988**	**4341**	**5693**	**5717**	**5720**	**5724**	**5725**	**5732**	**5733**	**5734**	**5735**	**5736**	**5737**	**5738**	**5739**	**5743**	**5746**
ΔIVs_gal_	0.3	0.3	**0.8**	**0.6**	0.2	**0.8**	**0.9**	**0.6**	0.3	**1.1**	**1.7**	**0.6**	**1.1**	0.3	**0.8**	0.4	0.3	0.3
ΔIIs_gal_	1.8	1.8	**5.6**	**3.0**	1.7	**6.2**	**5.5**	**4.0**	**3.3**	**2.9**	**4.3**	1.5	**2.9**	1.5	**2.6**	**2.5**	0.8	1.0
ΔIIs	9.4	9.3	**9.8**	9.4	9.0	**10.4**	**9.7**	**9.6**	9.4	**9.6**	**9.8**	9.3	**9.7**	9.2	**9.5**	**9.6**	9.1	9.3
ΔIs	61.7	61.8	**55.5**	**59.8**	62.8	**57.1**	**57.6**	**59.2**	**60.4**	**59.8**	**57.6**	61.9	**59.8**	61.9	**60.3**	**60.9**	63.0	62.7
MW (Da)	14,100	15,300	**13,900**	NP	NP	**13,200**	**13,900**	14,600	**13,100**	**13,200**	**13,000**	15,000	**13,600**	14,900	**13,300**	**13,400**	15,000	15,200
Anti-coag	194	198	192	197	204	**177**	194	194	**179**	185	186	205	191	196	191	188	199	201
Anti-FXa	NP	NP	NP	193	191	166	169	177	185	182	175	195	179	187	177	191	196	200
Anti-FIIa	NP	NP	NP	**177**	196	**147**	**164**	**174**	**170**	**178**	**163**	195	**167**	189	**174**	**179**	196	202
Ph. Eur. compliance	OK	OK	OK	OK	OK	OOS	OK	OK	OOS	OK	OK	OK	OK	OK	OK	OK	OK	OK

*Disaccharides w/w%; activities in U/mg on dried basis; NP, not performed; OOS, out-of-specification. Disaccharides w/w%; activities in units per mg of heparin on dry basis. Shaded cells indicate: ΔIVs_gal_>0.5; ΔIIs_gal_>2.0; ΔIIs>9.5; ΔIs < 61.0; MW < 14,000; anti-coag < 180; anti-FXa < 180; anti-FIIa < 180*.

*Ph. Eur. compliance at time of investigation (according to heparin sodium monograph Accessed 8 July 2013)*.

Conclusions drawn here result from data reported in this paper and additional historical data.

#### Disaccharide Analysis After Exhaustive Depolymerization

ΔIIs_gal_ contents of crude heparin batches from our different suppliers are periodically evaluated and used to build a database. Suppliers C and D have levels always lower than 1.0% whereas suppliers A and B have higher levels, with an average of ~2%. For pure heparin, the maximum ΔIIs_gal_ content found was 6.2% for batch 5720 (as previously indicated) from supplier A (Table 2). In contrast, the maximum ΔIIs_gal_ content found in crude heparin used to manufacture this batch was only 2.4%. Interestingly, although suppliers A and B have similar levels for crude heparin batches, pure heparin batches from supplier B had a maximum ΔIIs_gal_ content 2.0% compared with ~6% for supplier A. This shows that IIs_gal_ content can remain stable, or substantially increase during the purification of crude into pure heparin depending of the batches used, even when the same purification process is used. Disaccharide analysis of crude heparin is therefore not sufficient to predict the quality of the expected pure heparin.

**Table 2:** Disaccharide analysis of pure heparin and crude heparin batches.

**(a) From supplier A**.												
**Disaccharide, w/w or ratio %**	**Crude**	**Pure**	**Crude**	**Pure**	**Crude**	**Pure**	**Crude**	**Pure**	**Crude**	**Pure**	**Crude**	**Pure**
**Batch #**	**5717**	**5717**	**5720**	**5720**	**5724**	**5724**	**5732**	**5732**	**5734**	**5734**	**5743**	**5743**
ΔIIs_gal_ (h123)	0.9	1.7	2.3 (2.1–2.4)	6.2	2.2 (2.0–2.4)	5.5	1.5 (1.4–1.7)	3.3	1.5 (1.5–1.5)	4.3	1.1 (0.4–1.7)	0.8
ΔIIs (h123)	8.6	9.0	9.5 (9.1–9.7)	10.4	9.6 (9.5–9.7)	9.7	9.3 (9.2–9.4)	9.4	8.9 (8.8–9.0)	9.8	9.2 (9.0–9.3)	9.1
IIs_epoxy_/IIs_gal_ (h1)	125	44	327 (300–367)	55	274 (256–313)	52	320 (320–320)	47	252 (244–257)	59	179 (100–286)	80
IIs_epoxy_	1.1	0.8	7.4 (6.3–8.4)	3.4	6.1 (5.3–7.2)	2.9	4.8 (4.5–5.4)	1.5	3.8 (3.7–3.9)	2.5	2.0 (0.6–4.9)	0.6
IIs_epoxy_ + ΔIIs_gal_	2.0	2.5	9.7 (8.4–10.7)	9.6	8.3 (7.3–9.5)	8.4	6.3 (5.9–7.1)	4.8	5.3 (5.2–5.4)	6.8	3.1 (1.0–6.6)	1.4
**(b) From suppliers B, C and D**												
**Supplier**				**B**					**C**			**D**
**Disaccharide, w/w% or ratio**				**Crude**			**Pure**		**Pure**			**Pure**
**Batch #**				**3249**			**3429**		**3244**			**2744**
ΔIIs_gal_ (h123)				1.6			2.0		0.2			0.4
ΔIIs (h123)				9.4			9.3		9.3			9.5
IIs_epoxy_/IIs_gal_ (h1)				0			33		67			83
IIs_epoxy_				0.0			0.7		0.1			0.3
IIs_epoxy_ + ΔIIs_gal_				1.6			2.7		0.3			0.7

*h1, obtained after heparinase 1 depolymerisation and CTA/SAX analysis; h123, obtained after heparinase 1+2+3 depolymerisation and SAX analysis. Average values (typically of 3 lots; range is provided) for crude batches used to manufacture one pure heparin batch; batch number for this average crude batch is taken as same as corresponding pure batch*.

#### Evaluation of Total Degradation

IIs_epoxy_ content was obtained using a combination of both analyses (heparinases 1+2+3 and heparinase 1) as previously described. All results are reported (Table 2). Total degradation was evaluated by adding IIs_epoxy_ to ΔIIs_gal_.

#### Respective Importance of ΔIIs_gal_ and IIs_epoxy_ as 2-O Desulfation Markers in Pure and Crude Heparin Batches

In pure heparin obtained from crude from suppliers A, B, C, and D, IIs_epoxy_ was approximately half ΔIIs_gal_ content ($\frac{IIs_{epoxy}}{{\Delta IIs}_{gal}}$ between 33 and 83% [mean 58%]), whereas in crude heparin it varied from close to 0 for supplier B up to between 125 and 327% (mean 260%) for supplier A; thus making IIs_epoxy_ by far the most important 2-O desulfation marker in crude heparin from supplier A. In crude heparin, ΔIIs_gal_ therefore represented on average 100% of the total desulfation for supplier B, down to 28% for supplier A. As a consequence, ΔIIs_gal_ alone cannot be used *a priori* as a reliable marker to estimate the magnitude of crude heparin desulfation. In pure heparin samples, ΔIIs_gal_ was found to be a more reliable indicator due to the partial hydrolysis of IIs_epoxy_ into IIs_gal_ during the purification process used, thereby decreasing the bias observed when not taking into account IIs_epoxy_ species. The heparin purification process used hydrolyses most, but not all, IIs_epoxy_ to IIs_gal_.

The total amount of (IIs_epoxy_+IIs_gal_)% in crude batches was found similar to that in the derived pure heparin batches (Table 2). In addition to the absence of IIs_gal_ increase (supplier B, C, and D), this suggests that the manufacturing purification process used does not further 2-O desulfate heparin and that the increase of IIs_gal_ content in some batches therefore corresponds to transformation of IIs_epoxy_ into IIs_gal_ during the purification step. The difficulty of the identification of IIs_epoxy_ in crude heparin samples, as previously discussed, explains why the degradation may remain “hidden” in crude heparin.

#### Characterization of Desulfation Degradation Pathways in Heparin Samples

The maximum ΔIIs content observed in pure heparin was 10.4% (batch 5720; Table 1). This batch was derived from crude heparin batches with an initial content of 9.5% (Table 2A). The increase of iduronic acid (IIs, k3 pathway) was therefore limited, and the selectivity of the epoxide ring opening was mostly toward the formation of galacturonic acid (IIs_gal_, k2) (Figure 1). In pure heparin batch 5720 (Tables 1, 2A), IIs_epoxy_+IIs_gal_ reached 9.6% and IVs_gal_ 0.8%, indicating that >10% of the disaccharide units had been desulfated. This corresponds to the desulfation of ~3 disaccharide units per heparin chain containing an average 25 disaccharide units. There was no significant IIs_epoxy_ in crude heparin from supplier B (Table 2B), whereas IIs_gal_ was observed at a concentration of 1–2%, suggesting that the kinetics k1, k2, and k3 (Figure 1) greatly depend on the environment, i.e., the precise process conditions used when preparing crude heparin. Crude batches from suppliers C and D (results not shown) had (IIs_epoxy_+IIs_gal_) content lower than 1.0%, or very limited desulfation, further highlighting the differences in desulfation between different crude heparin suppliers.

**Figure 4:**
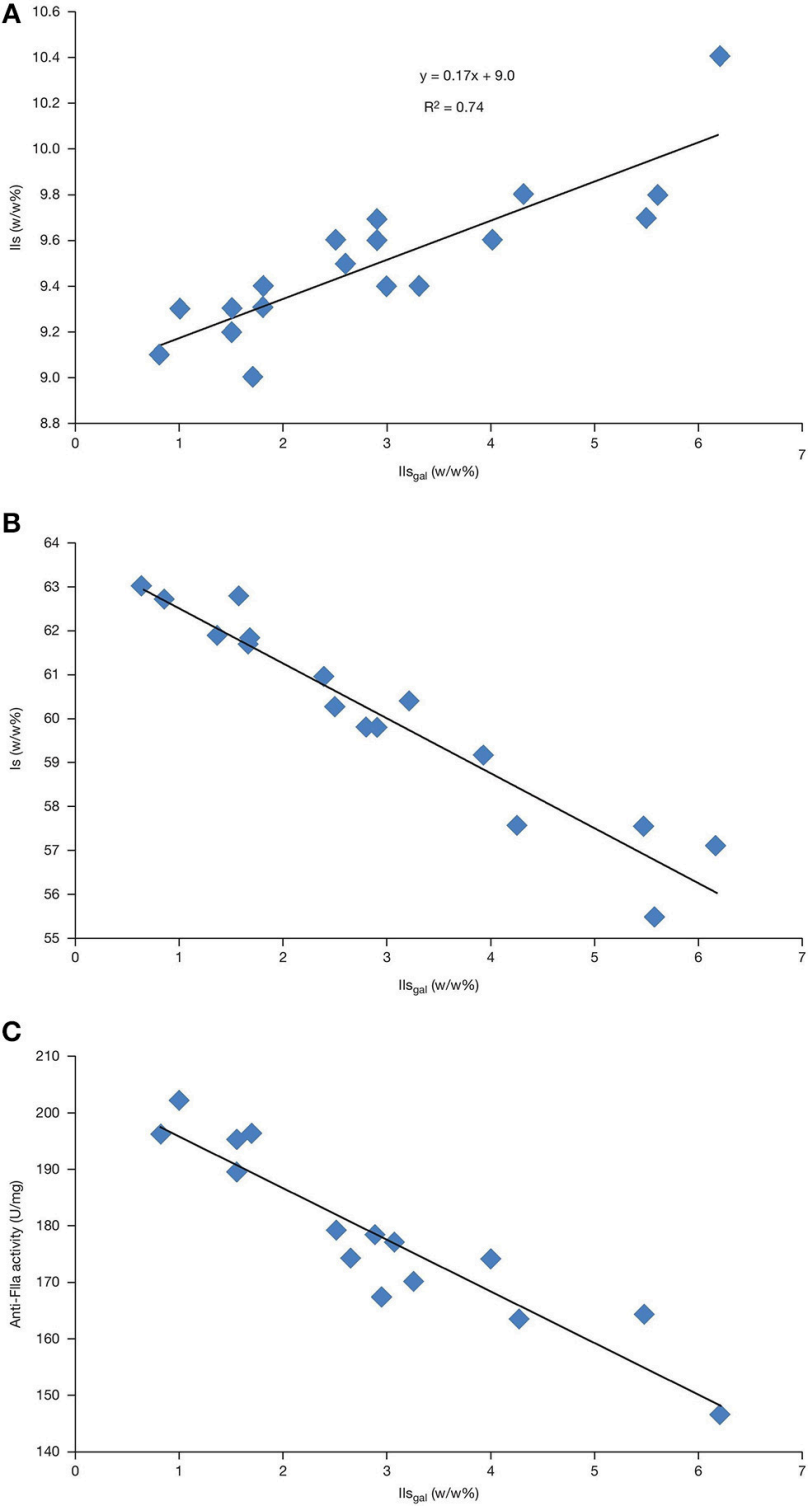
Analyses of pure heparin from supplier A showing the correlations between: **(A)** IIs and IIs_gal_; **(B)** Is and IIs_gal;_ and **(C)** anti-FIIa activity and IIs_gal_.

IIs and IIs_gal_ contents were correlated using data from supplier A samples in Table 1. The data confirmed that the preferred degradation pathway was toward IIs_gal_ with k2 = 6 × k3, 6 being the reciprocal of the slope (0.17) (Figure 4A). As expected, and due to the transformation of the former into the latter, Is negatively-correlated with IIs_gal_ (Figure 4B). Table 1 shows that the respective amounts of IIs_gal_ and IVs_gal_ (respectively 2.9 and 0.6% on average; $\frac{IIs_{gal}}{IVs_{gal}}$ ratio of 5) were not proportional to the content of their parent compounds, namely Is and IIIs (60.2 and 5.3% on average; $\frac{Is}{IIIs}$ ratio of 12). Indeed, IVs_gal_ content observed was approximately twice what would have been expected from the respective contents of Is and IIIs. As a consequence, IVs_gal_ should be monitored when performing a precise evaluation of 2-O desulfation.

#### Characterization of Relationship Between Desulfation, Anticoagulation, and Molecular Weight Measures in Pure Heparin Batches

The relationship between disaccharide analysis data, activities (including anti-coag, anti-FXa, and anti-FIIa activities), and MW were evaluated for pure heparin batches from supplier A. Results obtained are presented in Table 1. Compliance with the Ph. Eur. monograph specifications is also listed (2).

Anti-FIIa activity appeared to be the most sensitive assessment tool for differentiating between varying levels of desulfation. The activity (U/mg) ranking was generally anti-FIIa ≤ anti-FXa ≤ anti-coag. The anticoagulation data for the most desulfated batch (5720) suggested that such a batch could have passed the former Ph. Eur. anti-coag specification of 180 U/mg, yet having only 150 U/mg anti-FIIa activity. The OSCS crisis (22) illustrated the value of specifying individual activity requirements, more specifically anti-FIIa activity which is now required by the USP and Ph. Eur. Pharmacopeias. Anti-FIIa activity negatively correlated with IIs_gal_ levels, which could be explained by some structure–activity relationship (Figure 4C).

MW of the heparin batches ranged from 13,000 to 15,300 Da. Lower MW tended to be associated with lower anti-FIIa activity and vice versa; indeed, all batches with MW lower than 14,000 Da had anti-FIIa activity lower than 180 U/mg and all batches with MW higher than 15,000 Da were associated with anti-FIIa activity higher than 195 U/mg (Table 1). This indicated that some depolymerization occurred concomitantly with desulfation, which could explain the reduction in anti-FIIa activity. Hence, this is consistent with the requirement of chain lengths of at least 5,400 Da for triggering anti-FIIa activity. As a reminder, these absolute MW should not be directly compared with what could be obtained when using the current USP standard (not available at the time of this study).

Pure heparin batches obtained from crude batches of supplier A were expected to pass the 180 anti-FIIa U/mg USP (and Ph. Eur.) specification with ~4% desulfated disaccharides including ~2.5% (Figure 4C) IIs_gal_, ~1.3% (Table 2) IIs_epoxy_, and ~0.5% IVs_gal_ (Table 1) corresponding to an average of ~1 desulfated disaccharide per heparin chain. With another heparin supplier (pure heparin; data not presented), we could also link a rather low, although compliant anti-FIIa activity (≥180 U/mg) to the pattern of desulfation, up to a value of ~5%. Importantly our data indicate that, although all pure heparin batches show some evidence of desulfation, it is possible to obtain batches with very limited degradation indicating that these side reactions can be limited by appropriate process control.

## Conclusion

Crude heparin is a very complex mixture of variable purity with limited relevant release controls. With usual controls, poor intrinsic quality of the heparin may only be revealed after processing into pure heparin. This investigation demonstrated, through a relevant set of quality parameters, that desulfation reactions resulting from alkaline environment occur at varying magnitude during process conditions typically encountered during industrial purification of heparin out of mucosa. We further reported two chromatographic techniques that, applied after two different conditions of heparinase depolymerization, can be used to assess the entire extent of desulfation at the crude and pure heparin stages. We showed that IIs_gal_ and IIs_epoxy_ are the main non-endogenous disaccharides formed, the latter being potentially especially present in crude heparin due to its partial hydrolysis during the purification process, where it is particularly challenging to analyze. Although process degradation impurities observed during these studies are present at varying levels in all marketed pure heparin sources due to related process conditions, their control is not directly addressed within the different monographs; this is also the case for the other structural process impurities. Indeed, we showed that a heparin with 4% desulfation, or 1 modified disaccharide per chain, could pass the current anti-FIIa activity specification of the USP and Ph. Eur. monographs. To our knowledge, specific control of the process alteration of the heparin macromolecular backbone is not currently under consideration for future monograph evolution. Although disaccharide analysis is not systematically performed on each crude and pure heparin batch, our QC laboratory performs this analysis when qualifying a new supplier and when deemed justified on an at-risk basis (investigation, supplier control).

## Author Contributions

PA wrote the manuscript. CM performed the analyses. PM performed CTA-SAX analyses, contributed to and reviewed the manuscript. CV contributed to and reviewed the manuscript. All authors approved the submitted version.

### Conflict of Interest Statement

PA, CM, and PM were employees of Sanofi, Paris, France. CV is now an employee of Aspen, Notre Dame de Bondeville, France.

## Appendix

**Table:**

**ABBREVIATIONS**
AT	Antithrombin
BSA	Bovine serum albumin
Coag	Coagulant
CTA	Cetyltrimethylammonium
EMA	European Medicines Agency
FDA	Food and Drug Administration
FIIa	Factor IIa
FXa	Factor Xa
GAGs	Glycosaminoglycans
GPC	Gel permeation chromatography
HPLC	High-performance liquid chromatography
LMWH	Low-molecular-weight heparin
MW	Mass average molecular weight
NMR	Nuclear Magnetic Resonance spectroscopy
OOS	Out of specification
OSCS	Oversulfated chondroitin sulfate
Ph. Eur.	European Pharmacopoeia
SAX	Strong anion exchange
SEC	Size exclusion chromatography
UFH	Unfractionated heparin
USP	United States Pharmacopeia
UV	Ultraviolet
v/v	Volume/volume
w/w	Weight/weight
**Abbreviations for saccharide units and chemical modifications**	
U	Uronic acid
IdoA	L-iduronic acid
GlcA	D-glucuronic acid
ΔU	4,5-unsaturated uronic acid, eg ΔGlcA
ΔGlcA	4,5-unsaturated glucuronic acid
GlcN	D-glucosamine
NS	N-sulfate
NAc	N-acetyl
2S	2-O-sulfate
6S	6-O-sulfate
GalA	D-galacturonic acid
Epoxy	Epoxised iduronic acid
**Structural symbols**	
IVs_gal_	GalA-GlcNS
IIs_gal_	GalA-GlcNS,6S
IIs_epoxy_	GulA2,3epo -GlcNS,6S
IIs_glu_	GlcA-GlcNS,6S
IIIs_id_	IdoA-2S-GlcNS
IVs_glu_	GlcA-GlcNS
IIa_id_	IdoA-GlcNAc,6S
IIIa_id_	IdoA-2S-GlcNAc
Ia_id_	IdoA-2S-GlcNAc,6S
Is_id_	IdoA-2S-GlcNS,6S

The iduronic (id) or glucuronic (glu) structure of uronic acids is indicated for oligosaccharides, e.g., ΔIs-III_id_

Main disaccharides are in the following table. Underlined disaccharides have a 3-O sulfated glucosamine, e.g., IIs_glu_ (GlcA-GlcNS,3S,6S).

ΔIVa= ΔU-GlcNAc

ΔIVs_gal_= ΔGalA-GlcNS

ΔIVs= ΔU-GlcNS

ΔIIa= ΔU-GlcNAc,6S

ΔIIIa= ΔU2S-GlcNAc

ΔIIs_gal_= ΔGalA-GlcNS,6S

ΔIIs= ΔU-GlcNS,6S

ΔIIIs= ΔU2S-GlcNS

ΔIa= ΔU2S-GlcNAc,6S

ΔIIs= ΔU-GlcNS,3S,6S

ΔIs= ΔU2S-GlcNS,6S

ΔIs= ΔU2S-GlcNS,3S,6S
